# Commentary: Expert Opinion to “Imaging Bronchopulmonary Dysplasia—A Multimodality Update”

**DOI:** 10.3389/fmed.2021.737724

**Published:** 2021-10-21

**Authors:** Mark O. Wielpütz

**Affiliations:** ^1^Translational Lung Research Center (TLRC), German Center for Lung Research (DZL), University of Heidelberg, Heidelberg, Germany; ^2^Department of Diagnostic and Interventional Radiology, University of Heidelberg, Heidelberg, Germany; ^3^Department of Diagnostic and Interventional Radiology With Nuclear Medicine, Thoraxklinik at University of Heidelberg, Heidelberg, Germany

**Keywords:** magnetic resonance imaging, lung imaging, dysplasia, computed tomography, cystic fibrosis—CF, langerhans cell histiocytosis (LCH)

Chronic lung disease in infancy and early childhood poses a diagnostic challenge to medicine. (1) Entities are rare, with bronchopulmonary dysplasia (BPD) being the most prevalent in infancy and with increasing prevalence in grown-ups due to improved survival of prematurity. (2) Functional impairment is hard to measure because spirometry is practically impossible to perform at an early age. (3) Structural imaging may be a key component to diagnosis and monitoring, with computed tomography delivering high spatially resolved morphological information, but cumulative radiation dose and periprocedural efforts may be of high concern in radiation-susceptible young individuals and restrictive for imaging procedures (1). (4) Further, apart from definite diagnosis, therapeutic relevance should guide the decision to perform imaging tests and must be weighed against potential risks. Obviously, this somewhat trivial dogma holds true for all diagnostic tests in pediatric and adult clinical medicine, but it is specifically important in the research context where the generation of advances in knowledge and the testing of hypotheses may require a more liberal—yet well-considered—acquisition of study data, even in studies of human subjects. In the case of pediatric lung disease, imaging with structural and functional techniques may be the most comprehensive approach to characterize a pathophysiological process of the lungs and to study the natural evolution of findings and the changes induced by therapeutic intervention. In the case of new medication made available, imaging may provide a sensitive endpoint for measuring successful treatment (2).

Cystic fibrosis lung disease has been the most prominent example, for which sustained efforts in avoidance of radiation and improving imaging diagnostics have lead to the stepwise introduction of proton magnetic resonance imaging (MRI) for structural and functional lung disease, with a widely available MRI protocol, a scoring system for semi-quantification (3), cross-validation against CT (4), X-ray (5, 6), multiple breath wash-out (7) and spirometry (8), initial studies using MRI to monitor mid-term natural evolution (8), and as an endpoint for therapeutic intervention (6, 7, 9, 10). Of note, contrast-enhanced perfusion MRI is the first among many functional techniques to become widely available for the assessment of functional abnormalities in the CF lung (Figure 1) (11, 12). Besides, a multitude of novel functional techniques based on inhalational contrast agents such as hyperpolarized gas MRI using alternative nuclei, or non-contrast-dependent techniques such as Fourier Decomposition MRI and T1 mapping have driven deeper insight into structure-function relationships in cystic fibrosis lung disease (13–18). Logically, evidence from CF research as a model has been transferred to rarer pediatric lung diseases and vice versa, such as primary ciliary dyskinesia or BPD (19, 20). Although MRI has the advantage of relatively easy repeatability and “no dose,” its application in neonates and infants, especially in prematurity, may be cumbersome due to sedation, acquisition time, MRI-compatible equipment, lines, and drug delivery, and clinicians might demand a quick examination with highest structural resolution. Specific MRI scanners for neonates will probably remain a rare research tool in the very near future (21). In this case, “low dose” may be acceptable, and CT can play to its recent advantages in speed and clarity of details (22). Most recent works focus on the use of “ultra low dose” CT with a radiation dose close to a conventional X-ray, and will very quickly redefine the aforementioned case-by-case balance between the benefits and detriments of an imaging study.

**Figure 1:**
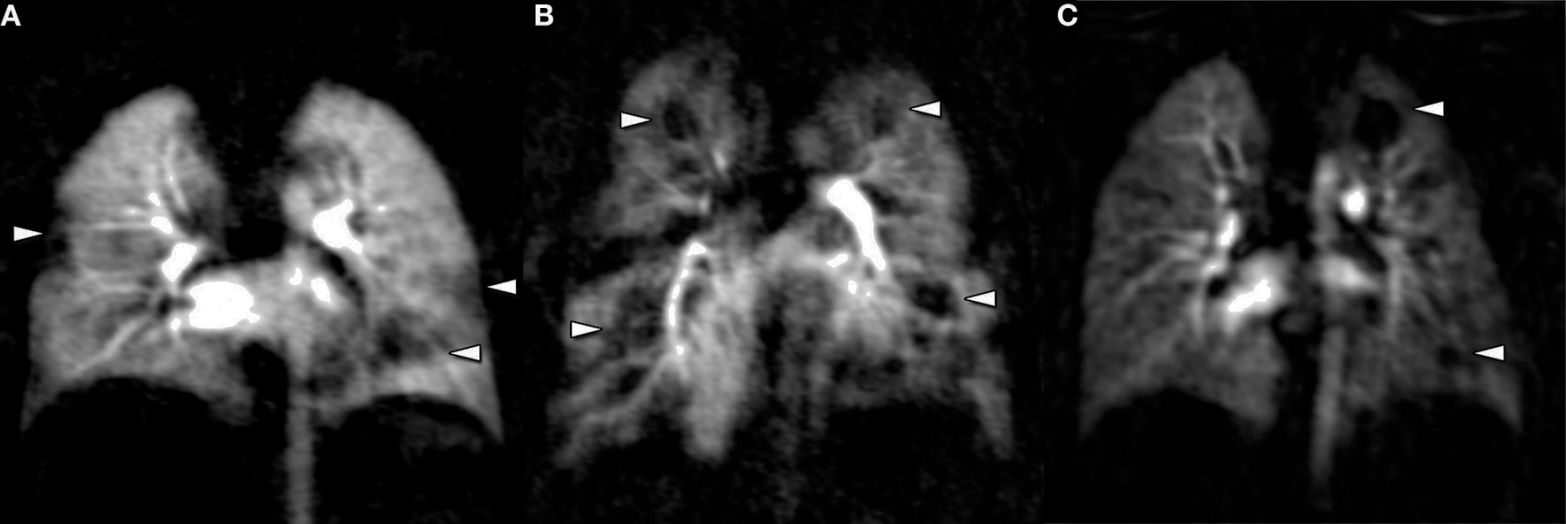
Perfusion MRI is a ready-to-use technique to study functional impairment in pediatric lung disease, based on gadolinium enhancement. Perfusion abnormalities can be found in conjunction with structural lung disease in cystic fibrosis **(A)**, Langerhans cell histiocytosis **(B)**, or bronchopulmonary dysplasia **(C)**. Of note, perfusion abnormalities are non-specific and must be reviewed together with morphological imaging studies.

At this point, the authors Semple et al. (23) step in and elucidate on the recent advancements in radiation dose reduction of CT techniques, and developments in MRI and lung ultrasound to study BPD in infants and young children, and bring in their personal long-standing experience in CF lung imaging research. They provide a short introduction into the growing clinical relevance of BPD in pediatric medicine, and why imaging will play an important role for diagnosis and follow-up, similar to what has been discussed in CF. With regard to CT in BPD, the most relevant developments are summarized concisely, offering the technical specifications applicable in routine medicine. The frontiers of research on quantitative imaging and implications for standardization of CT imaging parameters similar to other diagnostic tests are only briefly touched upon.

MRI is presented as a research tool for BPD, and most studies cited and discussed in the present manuscript refer to studies performed in CF patients, whereas actual evidence for MRI in BPD is relatively low, mostly due to the limitations mentioned above in very young children and high technical effort to achieve structural detail in their very small lungs. BPD as a disease is even detrimental to the conditions for lung MRI, because of regional tissue and thus proton loss (“minus pathology”) (24). Apart from the aforementioned neonate-specific prototype MRI (25), Förster et al. have added further BPD-specific evidence for the use of clinical state-of-the-art MRI for studying the pathophysiology of BPD in neonates. The authors found a decrease in T1 and an increase in T2 relaxation times were associated with an increased risk for BPD, which can partially be explained by tissue loss and perfusion changes in the diseased lung (26), and which was found also in COPD and CF (17, 27). Such findings may support the use of quantitative MRI for risk stratification and monitoring in BPD.

One should not forget about the potential of ultra-sound for pediatric lung imaging in the hands of experienced and ambitious examiners, which is also its main drawback—high levels of training required, and limited inter-observer agreement together with very limited overview render it an ancillary technique, and Semple et al. emphasize that in the context of BPD and intensive care treatment, X-ray remains the mainstay of everyday routine imaging to monitor lines and tubes, and also parenchymal lung changes.

## Author Contributions

The author confirms being the sole contributor of this work and has approved it for publication.

## Conflict of Interest

The author declares that the research was conducted in the absence of any commercial or financial relationships that could be construed as a potential conflict of interest.

## Publisher's Note

All claims expressed in this article are solely those of the authors and do not necessarily represent those of their affiliated organizations, or those of the publisher, the editors and the reviewers. Any product that may be evaluated in this article, or claim that may be made by its manufacturer, is not guaranteed or endorsed by the publisher.
